# A Clinical Comparative Study of Rectus Sheath Closure Techniques in Emergency Exploratory Laparotomy: Evaluating “Far-Near-Near-Far” vs. Conventional Closure Approach

**DOI:** 10.7759/cureus.45655

**Published:** 2023-09-20

**Authors:** Shreyash Garg, Moorat Singh Yadav, Kritika Singhal

**Affiliations:** 1 General Surgery, All India Institute of Medical Sciences, Bhopal, Bhopal, IND; 2 Community and Family Medicine, All India Institute of Medical Sciences, Bhopal, Bhopal, IND

**Keywords:** incisional hernia, wound dehiscence, continuous sutures, far-near-near-far suture, interrupted suture, exploratory laparotomy

## Abstract

Background: Midline exploratory laparotomy is essential in emergency surgery, and effective closure of the abdominal wall is crucial for optimal healing and reduced complications. The far-near-near-far technique for rectus sheath closure has gained attention due to potential advantages over conventional closure due to the fact that even if one suture gives way it does not affect the nearby suture and the rectus sheath is still held in place. This study aims to compare these techniques in emergency exploratory laparotomy.

Methods: This single-center prospective observational study included all patients undergoing emergency exploratory laparotomy at a tertiary care hospital. Patients were categorized into two groups based on the closure technique used: conventional closure or far-near-near-far technique. Sociodemographic data and comorbidities were collected. Operative time, closure time, and postoperative complications were assessed. Statistical analysis was performed using IBM SPSS Statistics 22.0 (IBM Corp., Armonk, NY).

Results: The study included patients with a mean age of 42.14 years. Operative and closure times did not significantly differ between the groups. There was no significant difference in comorbidities between the two techniques. The incidence of wound infection, dehiscence, burst abdomen, incisional hernia, and sinus formation did not significantly differ between the groups. Late complications were also similar.

Conclusion: The far-near-near-far technique for rectus sheath closure in emergency exploratory laparotomy showed comparable outcomes to conventional closure methods. No significant differences were found in operative time, closure time, or postoperative complications. This study contributes to the understanding of different closure techniques, allowing surgeons to make informed decisions

## Introduction

In emergency surgery, midline exploratory laparotomy is crucial in diagnosing and treating a wide range of life-threatening abdominal conditions [1]. The success of such procedures heavily relies on meticulous surgical techniques and the subsequent closure of the abdominal wall to promote optimal healing and reduce the risk of complications. The rectus sheath, a critical anatomical structure, has been the key focus of various closure techniques to enhance patient outcomes [2]. Among the numerous rectus sheath closure techniques, the far-near-near-far technique has gained significant attention in recent years due to its potential advantages over conventional closure methods.

Conventionally, abdominal wall closure involves a continuous suture technique or interrupted sutures placed in a layered fashion. However, concerns have been raised regarding these closures' strength, integrity, and durability, particularly in emergencies where the abdominal wall is often under increased tension due to trauma or infection. In contrast, the far-near-near-far technique offers an innovative approach that incorporates multiple sutures and distributes the tension more evenly, potentially minimizing the risk of wound dehiscence and incisional hernias [3]. A meta-analysis of 23 randomized trials showed that the odds of a burst are reduced to half with the interrupted closure method compared to the continuous process. In emergency surgery, interrupted sutures are better than the constant method as they have a “gigli saw” or “hack saw” effect [3].

This clinical comparison study aims to evaluate the outcomes associated with the far-near-near-far technique in comparison to conventional closure methods. Parameters such as time taken to operate, post-operative abdominal wall complications such as wound infection, dehiscence, burst abdomen, incisional hernia rates, and sinus formation were examined to assess the effectiveness of the far-near-near-far technique in an emergency exploratory laparotomy. Understanding the nuances of different closure techniques is vital for surgeons, enabling them to make informed decisions and employ the most appropriate method for each patient [4]. By shedding light on the clinical comparison of rectus sheath closure techniques, this article strives to contribute to the existing body of knowledge and enhance surgical practices in emergency abdominal surgery. Further, this article aims to provide a comprehensive clinical comparison between rectus sheath closure using the far-near-near-far technique and conventional closure in an emergency exploratory laparotomy.

## Materials and methods

Study design

It is a single-center prospective observational study conducted at a tertiary care hospital, AIIMS Bhopal.

Study setting

This study was conducted in the surgery and emergency department at All India Institute of Medical Sciences, Bhopal a tertiary care teaching hospital in Central India. The study was conducted from March 11, 2020 to December 2021.

Study population

All patients over 18 years of age undergoing emergency exploratory laparotomy procedures at AIIMS Bhopal between March 2020 to December 2021 were included in the study. Patients who underwent elective laparotomy, pre-operative proven malignancy, previous laparotomy for any condition (incisional hernia or burst abdomen), discharge against medical advice or death before the 10th post-operative day, any requirement of a re-laparotomy within 10 days for a cause not attributable to a complication of wound closure and lack of adequate post-operative follow-up were excluded from the study.

Sample size

This study incorporated universal sampling and included all the patients who underwent emergency exploratory laparotomy surgery in the surgery and emergency department at AIIMS Bhopal.

Ethical statement

Permission from the Institutional Human Ethics Committee- Post Graduate Research (IHEC-PGR) (LOP No: IHECPGRMD047) was taken before the commencement of the study. The subjects were informed about the research and procedures through a participant information sheet. Data collection was not done without written informed consent. Confidentiality of information has been maintained. Only the research team has access to personal information. Personal identifiers were removed during data analysis and reporting.

Data collection

Patients undergoing emergency exploratory laparotomy surgery from March 2020 to December 2021 who fulfilled the inclusion criteria were approached and informed consent was taken. Patients were included in two groups according to the surgeon's closure method, i.e., conventional closure or “far-near-near-far” technique of abdominal wall closure. An instrument was developed to obtain the demographic and clinical data of the subjects in the study. The instrument consisted of two parts: Demographic and Clinical data. The demographic data included the following information: age, sex, date of admission, date of operation, date of discharge, number of post-operative outpatient visits, and period of postoperative follow-up in the hospital. Clinical data included information about the patients’ health status: the presence of comorbid illness, lifestyle factors, the diagnosis of the patient, the focus of insult, the operation done, the stoma formed, admission in the surgical intensive care unit for monitoring/mechanical ventilation, postoperative complications and the period of follow-up in the out-patient department. The follow-up was for twenty-one months and outcomes included wound infection, wound dehiscence, burst abdomen, and late complications (sinus formation, Incisional hernia).

Operational definitions

Continuous/Conventional Closure

Conventional closure included closure of rectus fascia in a continuous fashion. The sutures were placed 1 cm from the edge of the Linea alba on both sides and a 1-cm distance was maintained between two adjacent sutures.

Far-Near-Near-Far/Interrupted Closure

This technique included suture approximation of the rectus sheath, in an interrupted fashion. The entry and exit of Polypropylene was 2 cm from the wound edges and 1 cm from the edge of Linea Alba on either side. The distance between two adjacent sutures was 1 cm.

Statistical analysis

Statistical analysis was carried out using statistical packages for IBM SPSS Statistics 22.0 for Windows (IBM Corp., Armonk, NY). Continuous and categorical variables were expressed as frequencies, means, and percentages, respectively. The chi-square test was used to compare categorical variables. Two-sided p-values were considered statistically significant at p<0.05. Logistic regression was done to assess the association of demographic variables, comorbidities, and lifestyle factors with wound infection and wound dehiscence.

## Results

**Figure 1:**
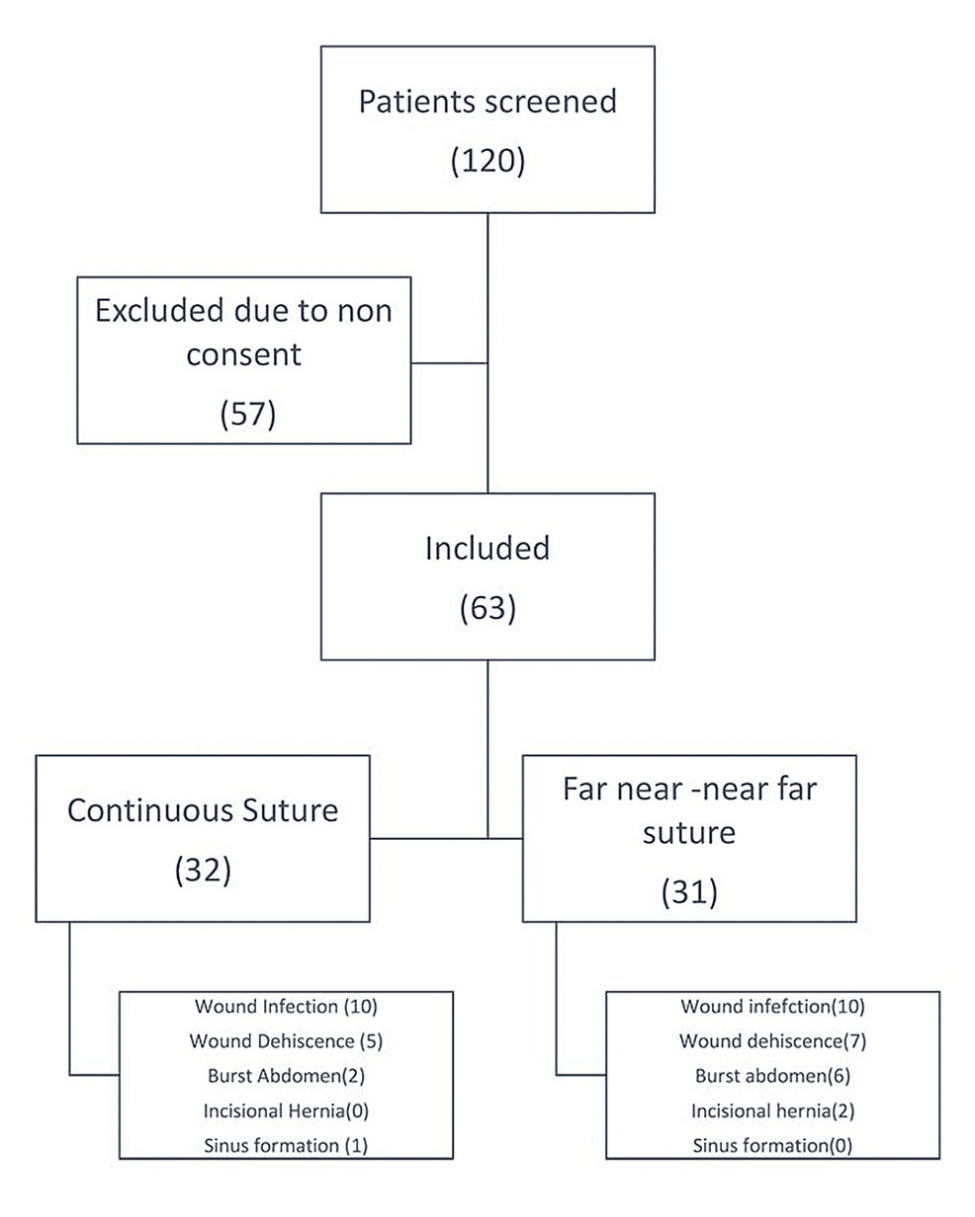
Flow of the study The number of participants at each stage are depicted in the flowchart.

The flow of the study is depicted in Figure 1. A total of 120 participants were screened and 63 were included for the study. There were 32 participants in the group with continuous sutures and 31 participants in the group with interrupted or far-near-near-far sutures.

The study revealed that the mean age of the subjects enrolled was 42.14 ± 15.12 years. The majority of subjects in the study were obese and males comprised 68.25% of the study population. Table 1 depicts the information on sociodemographic characteristics and comorbidities.

**Table 1:** Sociodemographic characteristics and comorbidities in participants p<0.05 was significant

		Interrupted (n=31)	Continuous (n=32)	P-value
Gender				
Male		23(74.2%)	20(62.5%)	0.319
Female		8(25.8%)	12(37.5%)	
Nutritional status				
Underweight		5(16.1%)	1(3.1%)	0.040
Normal		5(16.1%)	13(40.6%)	
Obese		21(67.7%)	18(56.3%)	
Lifestyle factors				
Smoker	Yes	9 (29.0%)	9 (28.1%)	0.936
	No	22 (71.0%)	23 (71.9%)	
Alcohol	Yes	11 (35.5%)	6 (18.7%)	0.135
	No	20 (64.5%)	26 (81.3%)	
Comorbidities				
Diabetes Mellitus	Yes	4(12.9%)	3(9.4%)	0.656
	No	27(87.1%)	29(90.6%)	
Hypertension	Yes	5(16.1%)	3(9.4%)	0.421
	No	26(83.9%)	29(90.6%)	
Jaundice	Yes	3(9.7%)	5(15.6%)	0.478
	No	28(90.3%)	27(84.4%)	
Renal failure	Yes	12(38.7%)	6(18.7%)	0.080
	No	19(61.3%)	26(81.3%)	
Pulmonary disease	Yes	2(6.5%)	1(3.1%)	0.535
	No	29(93.5%)	31(96.9%)	
Malignant disease	Yes	1(3.2%)	0(0.0%)	0.306
	No	30(96.8%)	32(100.0%)	
Anaemia	Yes	7 (22.6%)	5 (15.6%)	0.482
	No	24 (77.4%)	27 (84.4%)	
Hypoalbuminemia	Yes	16 (51.6%)	20 (62.5%)	0.383
	No	15 (48.4%)	12 (37.5%)	
Heart disease	Yes	2 (6.5%)	1 (3.1%)	0.535
	No	29 (93.5%)	31 (96.9%)	

The mean total operating time in the continuous group was 177.25 ± 45.88 minutes which was longer than that in the far-near-near-far group was 160.80 ± 45.27 minutes (p=0.157). The mean total time to close the wound in the continuous group was 16.34 ± 3.219 minutes which was slightly lesser than that in the far-near-near-far group 17.94 ± 3.54 minutes (p=0.067). The two groups matched in terms of comorbidities and lifestyle factors. 18.8% patients in the continuous group had a stoma made and required post-operative ICU admission in contrast to 41.9% patients in the far-near-near-far group (Table 2). 15.6% patients in the continuous group required mechanical ventilation post ICU admission while that in interrupted group were 35.5% (p=0.070).

**Table 2:** Additional Factors affecting post-operative abdominal wall complications in participants p<0.05 was significant; ICU: Intensive Care Unit

	Far-near-near-far (n=31)	Continuous (n=32)	P-value
Stoma			
Yes	13(41.9%)	6(18.8%)	0.045
No	18(58.1%)	26(81.3%)	
Post –operative ICU admission			
Yes	13(41.9%)	6(18.8%)	0.045
No	18(58.1%)	26(81.3%)	
Mechanical Ventilation			
Yes	11(35.5%)	5(15.6%)	0.070
No	20(64.5%)	27(84.4%)	

Table 3 depicts the post-op abdominal wall complications in the continuous and far-near-near-far group. The period of follow-up ranged from 10 days to 22 months with an average follow-up period of 11.11 months. The mean follow-up period in the far-near-near-far group was 6.82 ± 5.47 months while it was 9.97 ± 3.61 months in the continuous group (p=0.011). This time was considered sufficient to detect late wound complications.

**Table 3:** Comparison of post-operative abdominal wall complications between the groups p<0.05 was significant

		Far-near-near-far (n=31)	Continuous (n=32)	P-value
Wound Infection	Yes	10(32.3%)	10(31.3%)	0.932
	No	21(67.7%)	22(68.8%)	
Wound dehiscence	Yes	7(22.6%)	5(15.6%)	0.482
	No	24(77.4%)	27(84.4%)	
Burst abdomen	Yes	6(19.4%)	2(6.3%)	0.118
	No	25(80.6%)	30(93.8%)	
Incisional Hernia	Yes	2(6.5%)	0(0.0%)	0.144
	No	29(93.5%)	32(100.0%)	
Sinus formation	Yes	0(0.0%)	1(3.1%)	0.321
	No	31(100.0%)	31(96.9%)	

**Figure 2:**
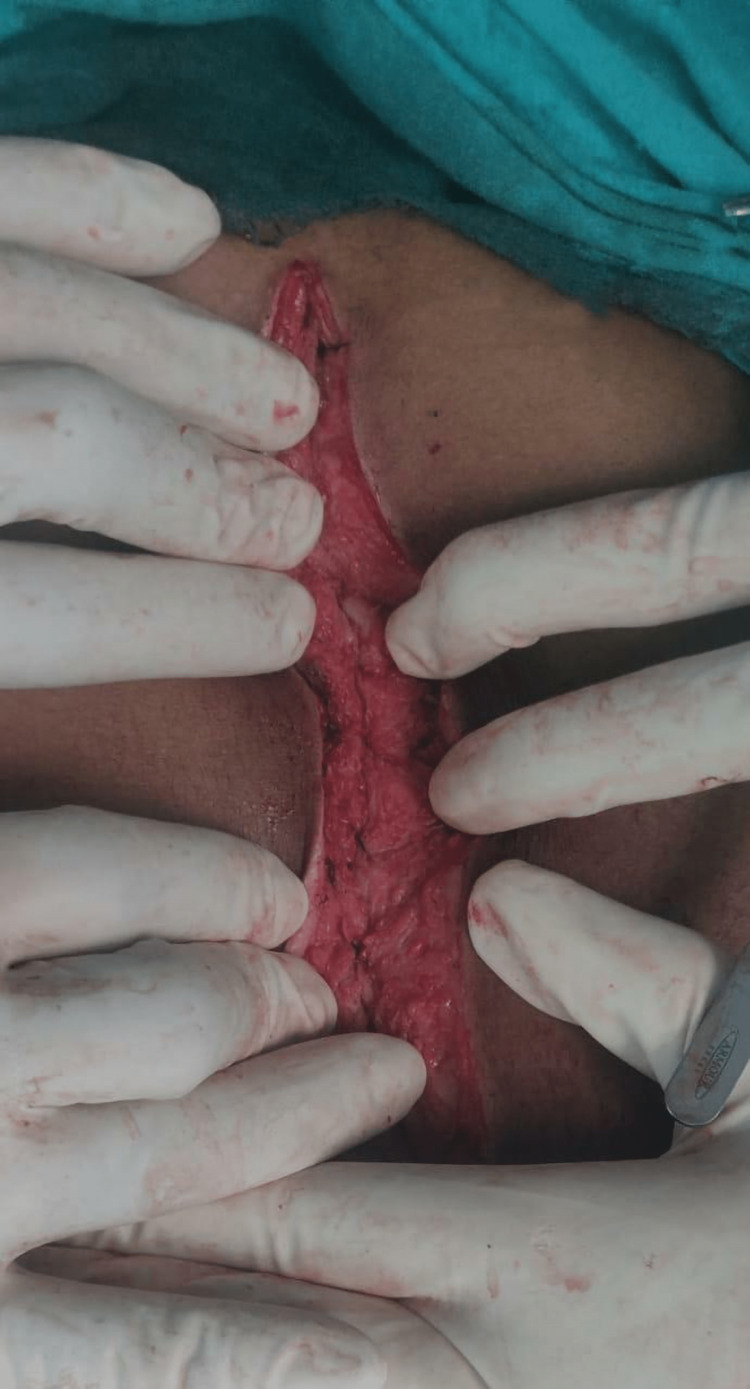
Continuous technique of rectus sheath closure Closure of rectus fascia in a continuous fashion, sutures were placed 1 cm from the Linea alba on both sides.

**Figure 3:**
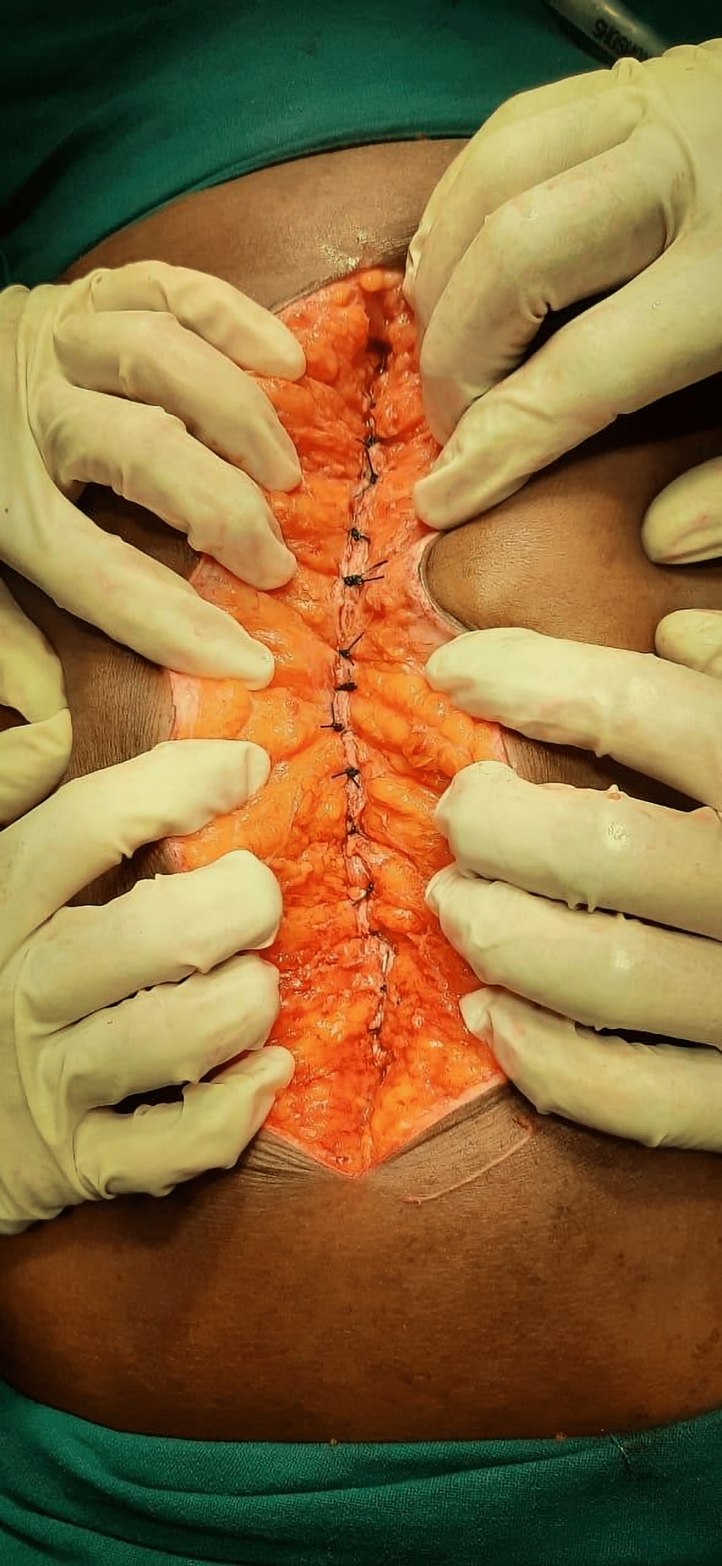
Far-near-near-far rectus sheath closure The entry and exit of Polypropylene were 2 cm from the wound edges and 1 cm from the edge of Linea alba on either side.

Table 4 shows that patients with hypoalbuminemia had a significantly increased risk (7.91 times greater) of developing wound infection with odds ratio 7.92 (CI 1.59-39.32) and p-value 0.011. Table 5 shows that patients with diabetes mellitus (DM), anemia and hypoalbuminemia had a significantly increased risk of developing wound dehiscence with an OR 59.81 (CI 1.59-39.32) and p-value 0.021 for DM, OR 30.79 (CI 1.29-733.6) and p-value 0.034 for anemia and OR 25.40 (CI 1.43-449.16) and p-value 0.027 for hypoalbuminemia. Perforation was the cause of acute abdomen in 57% of the cases and out of these gastro-duodenal perforation constituted 50% of perforations with 19.44% in continuous group and 30.55% in interrupted group. This was followed by ileum perforation in 19.43% of patients with 2.77% in continuous group and 16.66% in far-near-near-far group. Figures 2, 3 illustrate continuous and far-near-near-far type of rectus sheath closure, respectively.

## Discussion

The closure of the rectus sheath is critical in exploratory laparotomy, particularly in emergencies where the abdominal wall is under increased tension. The choice of closure technique can significantly impact patient outcomes, including the incidence of postoperative complications [5]. In this study, we compared the conventional rectus sheath closure with the far-near-near-far technique in an emergency exploratory laparotomy. We evaluated various parameters such as operative time, postoperative complications, and fascial closure time. The study's results provide essential insights into comparing the two closure techniques. The demographic characteristics of the study population, including age, gender, and body mass index (BMI), were analyzed to assess any potential differences between the continuous and interrupted technique groups. The mean age and BMI were slightly higher in the interrupted group, although these differences were not statistically significant. Gender distribution showed a higher percentage of males in both groups. Regarding operative factors, the mean total operating time did not differ significantly between the continuous and interrupted technique groups in contrast to other studies [6,7]. However, the mean full-time to close the wound was slightly higher in the interrupted group, although not statistically significant. These findings suggest that the far-near-near-far technique does not significantly prolong the overall operative time. However, it was noticed during the surgeries that surgeons preferred the continuous method more when the duration of surgery seemed to get stretched. The study also examined comorbidities and lifestyle factors among the study participants. The prevalence of various comorbidities, such as diabetes mellitus, hypertension, jaundice, and renal failure, was higher in the interrupted group compared to the continuous group. However, these differences were not statistically significant, indicating that specific comorbidities did not influence the choice of closure technique. Our findings demonstrate no statistically significant difference in the incidence of wound infection, wound dehiscence, burst abdomen, incisional hernia, and sinus formation between the two closure techniques. These results suggest that both the continuous and far-near-near-far techniques are comparable in their ability to prevent postoperative abdominal wall complications. A meta-analysis of 23 randomized trials showed that the odds of a burst are reduced to half with the interrupted closure method compared to the continuous process. In emergency surgery, interrupted sutures are better than the constant method as they have a “gigli saw” or “hack saw” effect [3]. In conventional abdominal closure, the primary advantage of layered closure is that as the individual fascial layer is sequentially closed, multiple strands exist so that if a break, the incision is held intact by the remaining sutures. At the same time, continuous fascial mass closure with a single closure allows the even tension distribution across the entire length of the suture, which minimizes tissue strangulation. However, if applied in layered closure, excessive tension leads to tissue necrosis and resultant closure failure [8,9]. Agrawal et al. have concluded that interrupted abdominal wall closure prevents burst abdomen in their randomized controlled trial comparing interrupted X and conventional continuous closures in surgical and gynecological patients [10,11]. However, it is essential to note that the sample size in our study was relatively small, and further research with a larger cohort is needed to validate these findings. Postoperative outcomes and complications were evaluated to assess the effectiveness of the closure techniques. The follow-up period ranged from 10 days to 22 months, providing a sufficient timeframe to detect even late wound complications, although 36 months is advised [12]. The far-near-near-far group exhibited a shorter mean follow-up period than the continuous group, which may have implications for assessing long-term complications. The study reported the incidence of postoperative complications such as wound infection, wound dehiscence, burst abdomen, and late complications (sinus formation and incisional hernia).

One of the far-near-far technique's key advantages is its ability to distribute tension more evenly across the abdominal wall, potentially reducing the risk of wound dehiscence and incisional hernias. The far-near-near-far technique of abdominal wall closure is advantageous because it prevents the simultaneous involvement of the entire sheath and the sutures due to interruptions. This means that if one suture is infected and loosens, it does not spread to the nearby suture, and because the tension is equally distributed amongst all the sutures, the rectus sheath is still in place. Interestingly, a vent is inadvertently created, allowing the collected from the affected site to drain through, helping other sutures stay intact. This benefits us, as easy closure can be planned later without planning a significant operation when the patient stabilizes. This has a substantial advantage over the continuous technique because if any of the bites of continuous suture give way, it weakens the entire fascial closure and makes early closures difficult [13]. However, our study did not show a significant difference in the incidence of these complications between the two closure techniques. This may be attributed to our study's limited sample size and the heterogeneity of patient characteristics. Future research with a larger cohort and more standardized patient selection criteria may provide more precise insights into the far-near-far technique's impact on wound healing and hernia formation. In our logistic regression analysis, hypoalbuminemia was significantly associated with an increased risk of wound infection. Hypoalbuminemia can weaken the immune system, impair tissue repair, and create an environment that is more conducive to infection [14]. In contrast, diabetes mellitus, anemia, and hypoalbuminemia were associated with an increased risk of wound dehiscence. These findings emphasize the importance of preoperative optimization of patients' nutritional status and management of comorbidities to minimize the risk of postoperative complications. Surgeons should consider these factors when selecting a closure technique and implementing perioperative care protocols.

While the study provides valuable information on the clinical comparison between the far-near-near-far technique and conventional closure methods, several limitations should be acknowledged. Firstly, the study design is observational, which may introduce bias and confounding factors, and the relatively small sample size limits the findings' generalizability. A randomized controlled trial with a larger sample size would provide more substantial evidence for comparing the two closure techniques. Secondly, the study was conducted at a single center, which may limit the generalizability of the findings. Multi-center studies involving diverse populations would enhance the external validity of the results. Additionally, the surgeon's discretion in selecting the closure technique introduces potential bias, and a randomized controlled trial would mitigate this bias. Finally, the far-near-near-far group exhibited a shorter mean follow-up period than the continuous group, which may have implications for assessing long-term complications.

## Conclusions

In conclusion, this study contributes to the existing knowledge on rectus sheath closure techniques in emergency exploratory laparotomy. Our study provides initial insights into the clinical comparison between conventional rectus sheath closure and the far-near-near-far process in an emergency exploratory laparotomy. While we did not observe significant differences in postoperative abdominal wall complications between the two approaches, further research with larger sample sizes is warranted. The far-near-near-far technique may offer potential advantages in terms of tension distribution and is balanced in terms of operative time in contrast to popular belief. Still, additional studies are needed to confirm these findings. Surgeons should consider individual patient characteristics and optimize perioperative care to minimize complications in emergency abdominal surgeries. This study adds valuable insights to the surgical community, highlighting the need for comprehensive assessment when selecting abdominal closure techniques in emergency abdominal surgery.

## Appendices

**Table 4:** Logistic regression model for wound infection Binomial logistic regression; *p<0.05 significant

Patient characteristics	Odds Ratio	P value	95% Confidence Interval	
			Lower	Upper
Age	1.056	0.055	0.999	1.117
Sex	0.165	0.053	0.027	1.027
Diabetes mellitus	7.009	0.155	0.479	102.654
Hypertension	1.078	0.951	0.097	12.047
Jaundice	2.666	0.393	0.281	25.281
Renal failure	0.299	0.163	0.055	1.632
Pulmonary disease	0.917	0.968	0.014	60.521
Malignant disease	0.000	1.000	0.000	0.000
Smoking	0.279	0.293	0.026	3.009
Alcohol consumption	1.977	0.555	0.206	19.021
Anemia	3.196	0.198	0.545	18.751
Hypo albumin	7.921	0.011*	1.595	39.328
Heart disease	0.465	0.761	0.003	64.586

**Table 5:** Logistic regression model for wound dehiscence Binomial logistic regression; *p<0.05 significant

Patient characteristics	Odds Ratio	P value	95% Confidence Interval	
			Lower	Upper
Age	1.071	0.086	0.990	1.159
Sex	0.156	0.228	0.008	3.202
Diabetes mellitus	59.819	0.021*	1.848	1936.008
Hypertension	0.716	0.828	0.035	14.556
Jaundice	10.291	0.138	0.474	223.548
Renal failure	0.952	0.965	0.109	8.352
Pulmonary disease	0.007	0.103	0.000	2.715
Malignant disease	0.000	1.000	0.000	.
Smoker	3.557	0.469	0.115	110.168
Alcohol	0.844	0.904	0.054	13.106
Anemia	30.799	0.034*	1.293	733.609
Hypo albumin	25.406	0.027*	1.437	449.167
Heart disease	6.717	0.381	0.095	476.331
